# Unlocking the dual healing powers of plant-based metallic nanoparticles: managing diabetes and tackling male infertility challenges

**DOI:** 10.3389/fendo.2025.1482127

**Published:** 2025-07-04

**Authors:** Ayesha Siddiqa, Rahmatullah Qureshi, Ghazala Yasmin, Shaista Rafique, Noor-Ul-Ain Zafar, Chudary Sadam Hussain, Sana ur Rehman, Neelum Naheed

**Affiliations:** ^1^ Department of Botany, Pir Mehr Ali Shah-Arid Agriculture University, Rawalpindi, Pakistan; ^2^ Department of Botany, Government Graduate College for Women, Jhelum, Pakistan; ^3^ National Research Center of Intercropping, The Islamia University of Bahawalpur, Bahawalpur, Pakistan

**Keywords:** diabetes mellitus, male infertility, nanotechnology, anti-diabetic drugs, phyto-nanoparticles

## Abstract

*Diabetes mellitus* (DM) is a severe metabolic disorder characterized by an increase in blood glucose level due to insufficient insulin production or failure of insulin action on targeted tissues or both. DM impacts male reproductive health across four aspects: ejaculation, erectile dysfunction, structural alterations in reproductive organs, and alterations in semen quality. The population of male individuals with diabetes is steadily rising, paralleled by an increase in fertility issues among men. A WHO report states that diabetes mellitus affects about 171 million (2.8%) persons worldwide. Anti-diabetic medications that are now on the market are expensive and have several negative effects, including cardiac, hepatic, and renal failure in diabetic patients. Keeping in view, this review emphasizes the limitations of currently used synthetic anti-diabetic drugs and provides the progress in the development of phytogenic metallic NPs (NP)in the treatment of diabetes and associated male infertility. To collect data, various databases were examined, including Springer Link, Google Scholar, PubMed, Wiley Online Library, and Science Direct. Several studies and research reports based on nanotechnological approaches in the formulation of anti-diabetic drugs have pointed out the fact that research in the formulation of nanodrugs has improved strategies for combating diabetes and associated male infertility based on the plausible molecular mechanism of action of the drugs. These nanodrugs have been observed to significantly influence regulatory mechanisms through their effects on pancreatic α-amylase, intestinal α-glucosidase, insulin action, and glucose uptake across various *in vivo* and *in vitro* systems. Moreover, integrating nanotechnological methodologies with the exploration of herbal compounds further enhances the understanding of their chemical potential. This synergistic approach may pave the way for identifying novel drug candidates with exceptional therapeutic efficacy, offering significant advantages in the management of diabetes and associated male infertility for the betterment of humanity. Furthermore, the personalized design of plant-based metallic NPs has the potential to significantly advance precision medicine techniques for the treatment of male infertility and diabetes.

## 1 Introduction

Diabetes mellitus (DM) is a serious metabolic disorder characterized by elevated blood glucose levels resulting from insufficient insulin production, impaired insulin action on target tissues, or a combination of both. It is a major risk factor for cardiovascular diseases, which account for approximately 50% of deaths among individuals with diabetes (1, 2). The prevalence of diabetes is increasing worldwide due to an increase in obesity and a sedentary lifestyle. In a report released by (World Health Organization in 2000, it was estimated that over 171 million (2.8%) people are living with diabetes mellitus throughout the world (3, 4). This number is expected to increase to 366 million (4.4%) by 2030. According to the International Diabetes Federation, the incidence of diabetes in Pakistan in 2016 (5, 6), 2018 (1, 7) and 2019 (8, 9) It was 11.77%, 16.98%, and 17.1%, respectively, and in 2022, the prevalence rate of adults was 26.7% in Pakistan (9, 10).

**Figure 1:**
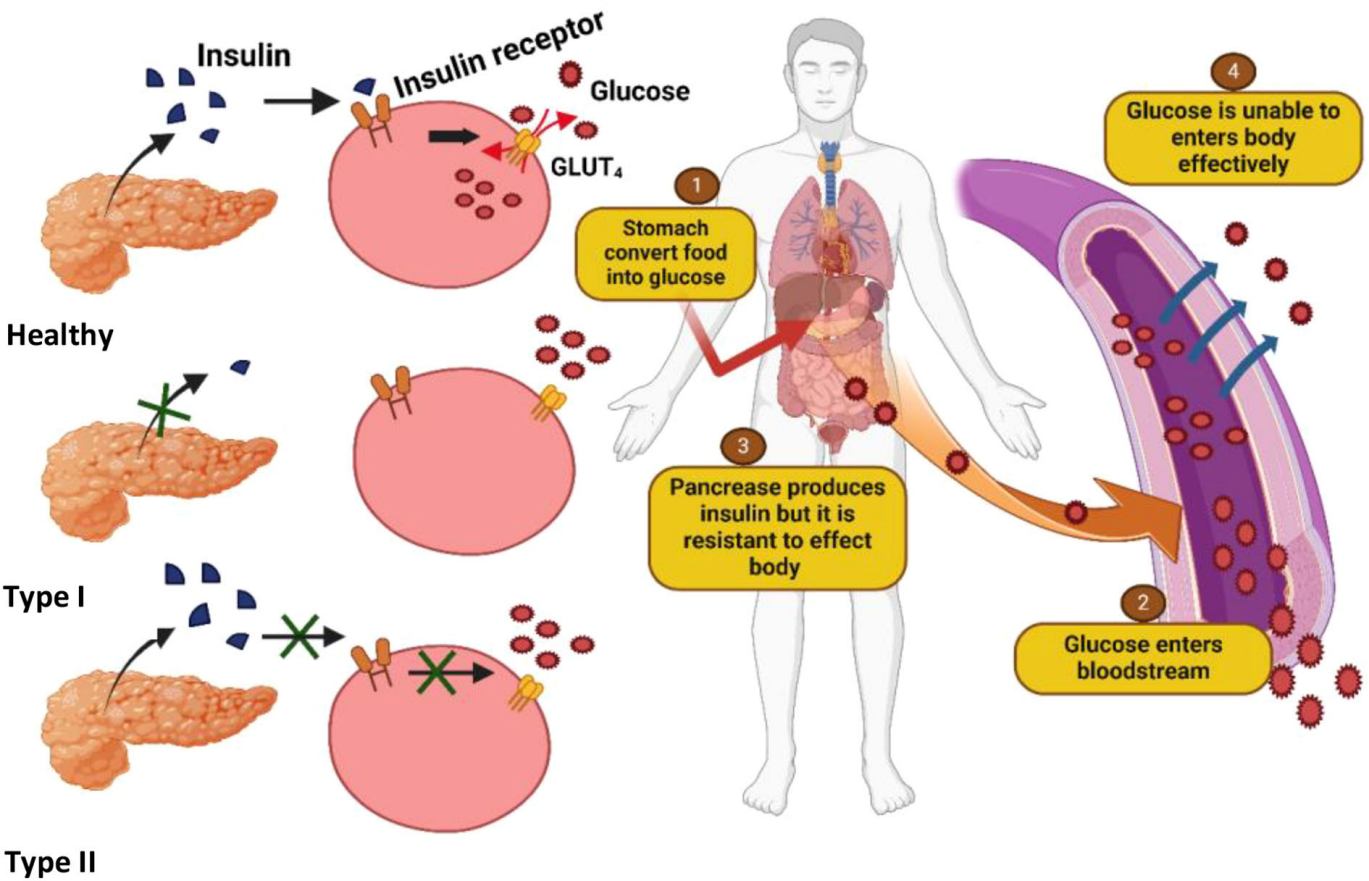
Illustration of differences in insulin production and glucose uptake among normal individuals, Type I diabetics (complete insulin deficiency), and Type II diabetics (insulin resistance and impaired uptake), leading to chronic hyperglycemia.

There are two primary forms of diabetes mellitus. Type 1 diabetes mellitus (T1DM), also known as insulin-dependent diabetes, is characterized by the autoimmune destruction of pancreatic beta cells, leading to a complete deficiency of insulin and resulting in chronic hyperglycemia (see **Figure 1**). Type 2 diabetes mellitus (T2DM), or non-insulin-dependent diabetes, is a metabolic disorder arising from either inadequate insulin production or the body’s inability to effectively utilize insulin, also culminating in elevated blood glucose levels (11). Dietary carbohydrates are broken down during digestion into glucose, which is subsequently absorbed through the walls of the small intestine into the bloodstream. Insulin, a hormone secreted by the pancreas, plays a critical role in facilitating the uptake of glucose into cells throughout the body, where it serves as a primary energy source.

In individuals with insulin resistance, however, cellular responsiveness to insulin is impaired, hindering glucose uptake and leading to elevated blood sugar levels. Initially, the pancreas compensates for insulin resistance by increasing insulin production; however, over time, this compensatory mechanism fails, leading to sustained hyperglycemia, which is a hallmark of type 2 diabetes (12) (**Figure 1**). This condition has become a significant public health concern, particularly in developing countries, where rapid urbanization and lifestyle changes, most notably the rising consumption of Western-style diets high in fats, have contributed to the growing prevalence of the disease. It is characterized by hyperglycemia, insulin resistance, and obesity. Obesity results in an imbalance between energy intake and energy expenditure. Besides obesity, it has a strong connection with dyslipidemia and hypertension. The interconnection between these conditions is a major risk factor for cardiovascular diseases (13, 14).

## 2 Diabetes associated male infertility and its underlying causes

The World Health Organization currently defines infertility as the inability of a sexually active couple (at least three times per month), not using contraception, to achieve pregnancy within one year. About 15% of sexually active couples are infertile (15, 16). And male factor infertility contributes to about 50% of the infertility cases (17). Research suggests that nearly half of male patients with diabetes experience decreased semen quality and impaired reproductive function. Diabetes-induced metabolic disorders can indeed have significant effects on male fertility and reproductive health (18). In recent years, increasing attention has been directed towards its effects on male reproductive health. Diabetes induces metabolic disturbances that contribute to oxidative stress, abnormal zinc metabolism, and insulin resistance (IR). These factors collectively impact male fertility and reproductive health.

**Figure 2:**
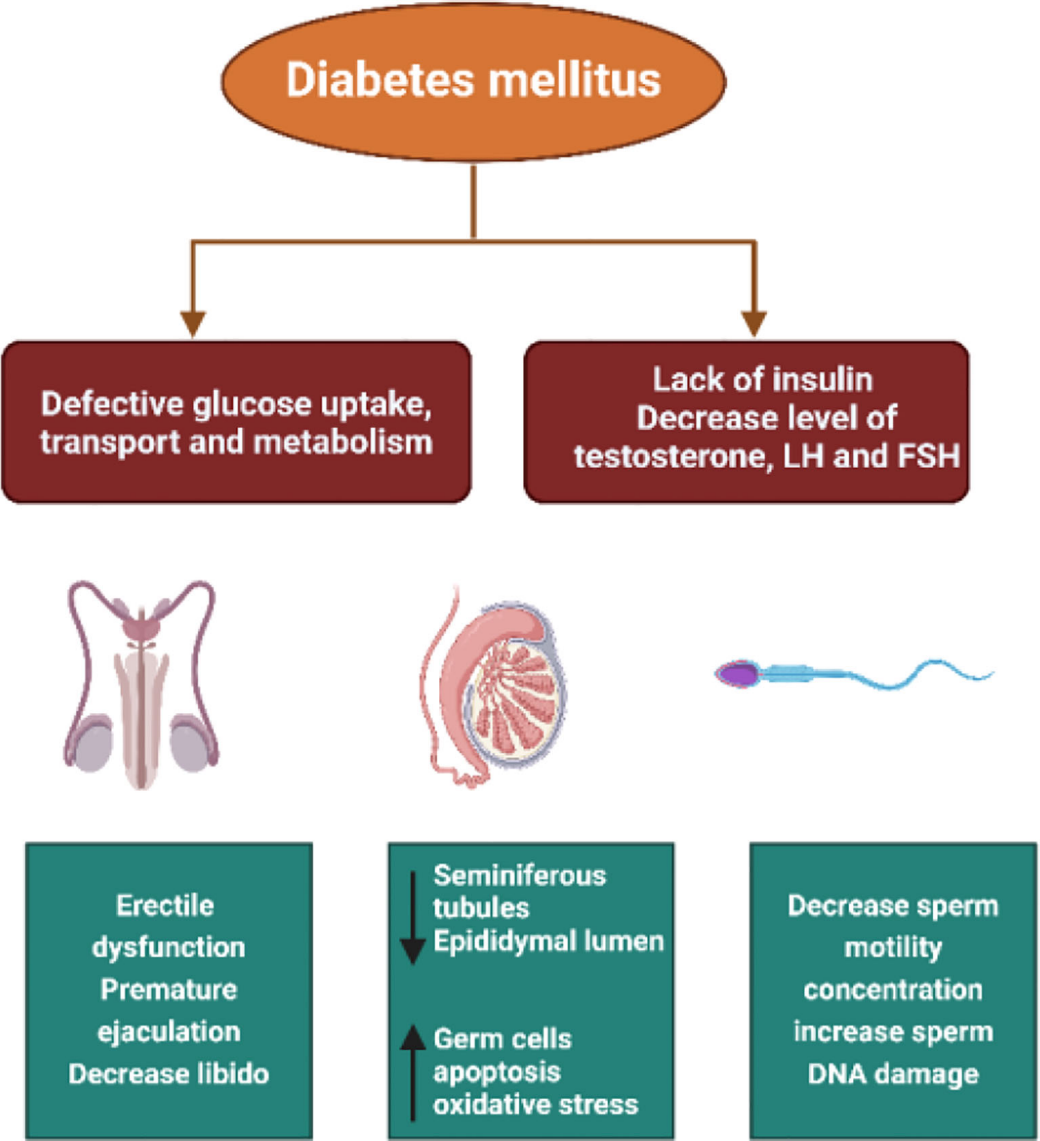
The pathways through which diabetes affects male fertility including oxidative stress, hormonal imbalance, impaired spermatogenesis, erectile dysfunction, and testicular structural damage.

Multiple studies conducted using animal models have demonstrated that DM significantly reduces fertility (19–21). This reduction is attributed to decreased sperm concentration and motility, increased seminal plasma abnormalities, and alterations in the normal morphology of sperm cells (22, 23). Additionally, patients with DM may experience other disturbances such as retrograde ejaculation, premature ejaculation, decreased libido, delayed sexual maturation, and impotence (15, 24–26) (**Figure 2**).

Some reproductive issues associated with diabetes are as follows

### 2.1 Erectile dysfunction

Among the various reproductive issues associated with diabetes, erectile dysfunction (ED) is a prominent concern. Studies indicate that 59% of diabetic men experience ED. The underlying cause often involves penile nerve thickening or beaded neuropathy. Additionally, decreased serum testosterone levels due to diabetes can negatively affect vascular endothelial function, further contributing to ED (27–32).

### 2.2 Testicular glucose metabolism and infertility

Hyperglycemia in diabetes alters testicular glucose metabolism. Glycogen, a key regulator of testicular development and spermatogenesis, plays a critical role by mobilizing glucose necessary for germ cell development. Disturbances in testicular carbohydrate metabolism, as observed in diabetes, can significantly contribute to testicular dysfunction and potentially lead to male infertility (33–35).

### 2.3 Structural and functional changes in reproductive organs

Diabetes induces structural and functional alterations in male reproductive organs. Notably, testicular blood flow velocity decreases, possibly attributed to reduced vascular endothelial growth factor (VEGF) expression (36, 37). Microcirculation disturbances result in testicular morphological and structural changes. In immature rats, diabetes can delay gonadal development, decrease sexual behavior, testosterone synthesis, and promote gonadal atrophy (36, 38). Seminiferous tubules (STs) may show signs of atrophy, thinning of the spermatogenic epithelium, and an increased presence of empty tubules. Some STs may contain multinucleated cells with two or three nuclei, along with evidence of enhanced vascular degeneration and germ cell apoptosis. Sertoli cells, located near the lumen of the STs, may accumulate cytoplasmic debris, and their ultrastructure often reveals irregular basement membranes and a reduced cell population. Structural alterations are also observed in Leydig cells, including irregularly shaped nuclei, abundant heterochromatin, lipid droplets, as well as damaged mitochondria and changes in the endoplasmic reticulum (39).

### 2.4 Histopathological changes in the testis

Studies investigating male reproductive dysfunction in the context of diabetes primarily focus on changes in testicular morphology. Data from various studies show reductions in the mass of different regions of the testis and a diminished sperm count in testicular tubules. Additionally, hyperglycemia can lead to histological alterations in the epididymal duct, including decreased germ cell populations, reduced stereocilia, clustering of epithelial cells, lipid vacuolization, and inflammation (40). Other histological changes observed in the testis tissue due to diabetes include wrinkled secretory epithelial cells in the prostate, testicular stromal hypertrophy, inflamed cells, prostatic intraepithelial neoplasia, and expanded secretory organelles. Reports in diabetic rodents also demonstrate reductions in the weight of seminal vesicles, as well as decreased weight and mass of testicular tissue. Moreover, the number of Leydig cells decreases, the seminiferous tubules’ diameter and germinal epithelium height decrease, and the volume of interstitial matrix increases in diabetic conditions (41, 42).

Reduced sperm quality is a recognized issue in diabetes, particularly in cases of T1DM. This is linked to various factors, including altered gene expression related to sperm DNA repair, mitochondrial DNA deletions, and decreased sperm motility (43, 44). Insulin levels in the bloodstream have been found to impact the acrosome and plasma membrane of sperm. Diabetic patients often experience reduced sperm motility and abnormal sperm morphology, which can impact fertility.

Hyperglycemia, a common feature of diabetes, affects all stages of spermatogenesis, including spermatogonia proliferation, spermatocyte division, and spermiogenesis. Research has consistently shown that diabetes can lead to decreased sperm count, motility, semen volume, and abnormal sperm morphology. However, some studies suggest that insulin therapy can improve sperm content and motility, while others find that semen volume may or may not be affected by diabetes (45). Diabetes can also cause damage to sperm DNA structure, potentially leading to infertility. Fortunately, controlling blood glucose levels has been shown to restore sperm numbers and motility in diabetic animal models. Normal lipid metabolism is essential for spermatogenesis, and hyperglycemia can disrupt this process, affecting triglyceride hydrolysis, cholesterol esters, and steroid hormones (40).

Hypogonadism, characterized by low testosterone levels, is another issue seen in diabetic men. Testosterone is crucial for male reproductive tissue development, sperm production, and overall sexual health (46, 47). Testosterone deficiency is more common in diabetic men, especially those over 40, and it may contribute to complications associated with diabetes (48). Testicular tissue degradation and small testes size have also been linked to low testosterone levels in diabetic men, impacting fertility and sexual function (49, 50).

### 2.5 Molecular mechanisms linking glucose metabolism and male reproductive dysfunction in diabetes

Male fertility is affected by Diabetes-induced dysregulation of glucose metabolism through several interconnected molecular pathways:

#### 2.5.1 Pancreatic α-amylase and intestinal α-glucosidase

These enzymes break down complex carbs into glucose, inhibiting their function and lowering hyperglycemia after meals. Prolonged hyperglycemia encourages oxidative stress and glycation end products (AGEs), which harm the seminiferous epithelium, disrupt the function of Leydig and Sertoli cells, and fragment sperm DNA (51).

#### 2.5.2 Insulin action and insulin receptors in testicular tissue

Sertoli and Leydig cells have receptors for insulin and insulin-like growth factor-1 (IGF-1). Insulin resistance impairs testosterone synthesis, breaks down the blood-testis barrier, and decreases spermatogenesis, all of which decrease these cells’ responsiveness. This is partially mediated by the MAPK and PI3K/Akt pathways, which are implicated in cell survival and glucose uptake (52).

#### 2.5.3 Glucose uptake and spermatogenic support

Spermatogenesis depends on Sertoli cells’ uptake of glucose (via the GLUT1 and GLUT3 transporters). The expression of the glucose transporter is dysregulated in diabetic conditions, which causes seminiferous tubules to lose energy. The maturation of germ cells is restricted, and apoptosis is increased (53).

#### 2.5.4 Oxidative stress and inflammation

Prolonged hyperglycemia weakens antioxidant defenses and produces excessive ROS. This oxidative imbalance results in decreased sperm motility, mitochondrial failure, and lipid peroxidation in sperm membranes. Inflammatory cytokines, such as TNF-α and IL-6, also affect the endocrine and exocrine processes of the testicles (54).

#### 2.5.5 Hormonal imbalance

Hypogonadotropic hypogonadism is linked to insulin resistance. It inhibits testosterone synthesis and Sertoli cell support for growing germ cells by decreasing LH and FSH signaling (55).

## 3 Current diabetic management strategies and their side effects

There are five classes of oral diabetes drugs (OHDs) available that function through four different pathways:

a. Improving insulin secretion in the pancreas (sulfonylurea & non-sulfonylurea)
b. Reducing glucose release from the liver (biguanides)
c. Lowering gastrointestinal absorption of carbohydrates (α-glucosidase inhibitor)
d. enhancing peripheral glucose disposal (biguanides and thiazolidinedione) (56, 57)

All of the medications have side effects (58, 59). Though it is essential to achieve glucose management as soon as possible to reduce the impact of glucose toxic effects, it is also vital to provide treatment to control other associated risks, such as oxidative stress, dyslipidemia, mitochondrial dysfunction, vascular complications, and so on (**Table 1**) (64–67). Diabetes cannot be cured totally, but its severity and symptoms can be managed with medications and lifestyle changes (68). Thiazolidinediones, Biguanides, Sulfonylureas, meglitinides, α-Glucosidase inhibitors, and Dipeptidyl peptidase-4 (DPP-4) inhibitors. These are some of the most regularly utilized pharmacological medications for the treatment of diabetes. These medicines are administered as the first line of defense to prevent the diabetic state from deteriorating (68, 69).

**Table 1:** A list of the current medications for type 2 diabetes mellitus, along with their disadvantages (44, 48, 60–63).

Class and examples	Dosing	Mechanism of action	Physiological effects	Glucose lowering efficacy	Disadvantages	Cost
Sulfonylureas (1965) Glyburide Glimepiride Glipizide Gliclazide	Once a day Or Twice a day	It binds to SUR1 on beta cells, closing KATP channels, depolarizing them, and allowing calcium influx.	Promote insulin secretion	high efficacy	Obesity Hypoglycaemia Need for Self-Monitoring of Blood Glucose Dosage titration	Low price
Biguanides (1957) Metformin	Once a day twice a day	AMP-activated protein kinase (AMPK) activation improves intestinal glucose regulation, reduces respiratory chain activity, and enhances cellular insulin signaling.	Reduce liver glucose production, improve insulin sensitivity, and increase Glucagon-like peptide-1 GLP-1 levels.	High efficacy	Gastrointestinal side effects Various potential contraindications, particularly involving renal dysfunction and hypoxemia	Low price
α-Glucosidase inhibitors (1995) Miglitol Voglibose Acarobose	Three times a day with meals	Blocking the activity of α-glucosidase In the gut	Prolonged glycemic response	Un assuming	Digestive problems	Normal price
Meglitinides (1997) Repaglinide	Taken with meals	SUR1 can bind to β cells. Their mode of action is more rapid and short in duration than compared of sulfonylureas	raise the secretion of insulin	From moderate to high	Increase in Weight Low blood sugar	Normal price
Thiazolidinediones (1997) Rosiglitazone Pioglitazone	Once a day	Agonists: Peroxisome proliferator-activated receptor gamma (PPAR-γ)	Greater insulin sensitivity Lower production of free fatty acids	High efficacy	Weight increase Accumulation of excess fluid in body tissues leads to swelling Heart failure	Low price
DPP-4 inhibitors (2006) Sitagliptin Saxagliptin Linagliptin Alogliptin Vildagliptin	Once a day Or Twice a day	Hinder the activity of DPP-4, and endogenous incretin levels increase	Insulin production increases glucose-dependently, while glucagon is inhibited.	Midway efficacy	Unestablished long-term safety Heighten the likelihood of pancreatic inflammation. Use of vildagliptin causes an increased risk of liver illness	High price
SGLT2 inhibitors (2012) Dapagliflozin Canagliflozin empagliflozin	Once a day	In proximal renal tubules, SGLT2 activity inhibits	Glucose excretion in the urine increases	Low to high efficacy	Unestablished long-term safety connection to urinary tract infections and perhaps genital diseases Osmotic diuresis may increase the risk of falls and hypotension. The risk of fractures increases Risk of an increase in diabetic ketoacidosis	High price
dopamine-2 agonist (2009) bromocriptine	Once a day	Hypothalamic dopamine receptors activate	Retention of hepatic glucose output Glucose disposal increases	Normal efficacy	Fatigue Nausea Dizziness	High price
Bile-acid sequestrant (2008) colesevelam	Once a day Or Twice a day	Hepatic bile salt production increases GLP1 secretion increases Liver farnesoid receptors activate	Hepatic glucose output is possible to decrease, and incretin secretion increases	Normal efficacy	Triglyceride levels increase Absorption of some other drugs increases Constipation	High price
insulin (1920s) Speedy acting (aspart, lispro, glulisine) Low acting (humulin-S, inuman rapid, actrapid) Intermediary acting (insulin, insulin basal) Long-term acting (glargine, detemir)	Once a day, four times a day	Insulin receptors are directly activated	Glucose disposal increases Hepatic glucose output lessens Lipolysis reduces	High efficacy	Weight increase Edema Hypoglycaemia	Varying
GLP-1RAs (2005) Liraglutide Lixisenatide Albiglutide Dulaglutide Exenatide	Once a day Or Twice a day	The GLP-1 receptor activates	Insulin production rises glucose-dependently, while glucagon secretion is inhibited decrease in excretion after eating enhance satiety	High efficacy	Unestablished long-term safety Pancreatitis Gastrointestinal issues Insertable	High price

### 3.1 Thiazolidinediones

Thiazolidinediones or ‘glitazones’ are a novel class of oral diabetes medications. Thiazolidinediones are insulin sensitizers that function mostly through increasing insulin sensitivity in target organs such as the liver and muscles (70). Pioglitazone, rosiglitazone, and troglitazone are thiazolidinedione-derived drugs. Troglitazone became available in 1997 shortly thereafter removed due to toxicity to the liver (62, 71).

#### 3.1.1 Mode of action

The activation of a transcription factor, peroxisome proliferator-activated receptor (PPAR), is the mechanism by which TZDs exert their anti-diabetic action. This factor affects the transcription of various genes involved in glucose and lipid metabolism and energy balance, such as fatty acyl-CoA synthase, malic enzyme, glucokinase, and glucose transporter 4 (GLUT4), among others. As a result, TZDs decrease insulin resistance (IR) in adipose tissue, muscle, and the liver (70, 71).

#### 3.1.2 Adverse effects of using thiazolidinediones

One of the main adverse effects associated with PPAR receptor activation is the enhanced proliferation of peripheral adipocytes, which leads to increased uptake of free fatty acids. This process can ultimately result in weight gain and an increase in peripheral fat mass (72, 73). Several recent studies and analyses have indicated the potential role of TZD in cardiovascular events in type 2 diabetic patients. In this way, meta-analyses of adverse outcomes from controlled studies have revealed a possible link between thiazolidinedione use and an elevated risk of ischemic myocardial events in diabetes patients (74, 75). Fluid retention is another known side effect associated with the use of thiazolidinediones (TZDs). It is hypothesized that TZD-induced renal edema results from the activation of sodium-coupled bicarbonate reabsorption in the renal proximal tubules. This leads to increased salt and water reabsorption, ultimately causing an expansion in kidney volume (76). The outcomes of these studies caused disagreement as well as confusion about the use of TZDs in diabetic treatment methods (68, 71).

### 3.2 Biguanide

Biguanides are a pharmacological and pharmaceutical class that relies on the biguanidine molecule (77, 78). These chemicals were first isolated from the plant Galega officinalis (79, 80). Guanidine, the active component of Galega officinalis, was demonstrated to reduce glucose levels in the 1920s and was used for synthesizing many anti-diabetic drugs (78, 81). They are known as another type of insulin sensitizer, and metformin is one of the most commonly used medications for diabetes in this class (68, 72). Biguanides do not act as true hypoglycemic agents (82, 83). Thiazolidinediones (TZDs) lower elevated blood glucose levels in patients with non-insulin dependent diabetes mellitus (NIDDM), but they do not significantly reduce blood glucose levels in non-diabetic individuals unless there has been prolonged fasting (84–89). Biguanides, compared to sulfonylureas, do not increase insulin secretion from p-cells; however, biguanide therapy may result in decreased insulin levels (89, 90).

#### 3.2.1 Adverse effects of biguanide

Symptoms of the gastrointestinal tract (e.g., nausea, vomiting, diarrhea, and abdominal discomfort) are among the side effects linked with the therapeutic use of all biguanides (78, 91). Metformin is seldom associated with acute hepatitis and cholestasis (81, 92–100) Metformin has been linked to malabsorption syndromes, which can result in electrolyte imbalances and vitamin B12 insufficiency (13, 81, 84–86, 90, 101–112) Diarrhea is associated with hypomagnesaemia, hypocalcemia, and hypokalemia (85, 104, 105, 113). Vitamin B12 deficiency can lead to megaloblastic anemia and various neuropathies. The underlying causes of this deficiency are not fully understood but appear to be multifactorial. Contributing factors may include alterations in gut microbiota, reduced gastrointestinal motility, competitive inhibition of B12 absorption, and disruptions in calcium-dependent membrane transport mechanisms in the terminal ileum (106, 113).

Lactic acidosis is an uncommon but possibly hazardous metformin adverse effect. The occurrence of this consequence is quite low: one case per 100,000 individuals receiving treatment (114–119). Lactic acidosis can be produced by very high metformin levels in blood vessels or by any circumstance that causes hypoxia or hepatic insufficiency, restricting the capacity of the body to break down lactate (120). Lactic acidosis usually arises in patients who have persisted in using metformin despite risks (114, 120). Renal insufficiency, indicated by serum creatinine levels of 1.5 mg/dL or higher in men and 1.4 mg/dL or higher in women, is a contraindication for metformin therapy. Additionally, conditions such as severe cardiac or pulmonary insufficiency that lead to reduced peripheral perfusion, lactic acidosis, liver disease, alcohol dependence, or the use of intramuscular contrast agents for radiographic imaging also represent exclusion criteria due to the increased risk of adverse effects, particularly lactic acidosis (80, 121).

### 3.3 Sulfonylureas

Sulfonylureas are categorized as either first-generation (e.g., tolbutamide and chlorpropamide) or second-generation (e.g., glyburide, gliclazide, glipizide, and glimepiride) (122, 123). Similar to first-generation sulfonylureas (such as tolbutamide, acetohexamide, and chlorpropamide), second-generation sulfonylureas also effectively lower hyperglycemia (124, 125). Second-generation sulfonylureas are preferred over first-generation agents due to their higher potency and more favorable safety profile. First-generation sulfonylureas are associated with a greater risk of adverse effects, including hypoglycemia, weight gain, and fluid retention, making second-generation drugs the recommended choice in clinical practice (126, 127).

#### 3.3.1 Mode of action

Sulfonylureas act by inhibiting the ATP-sensitive K channel (KATP), causing the release of insulin from the cells of the pancreas and therefore lowering blood glucose levels (128–131). Over 90 percent of sulfonylureas in the blood are linked to plasma proteins, causing interactions between drugs with salicylates, sulfonamides, and warfarin (123, 132). While the effectiveness of sulfonylureas varies, they tend to reduce A1C to a comparable extent as metformin, by 1.5 percentage points (117, 133).

#### 3.3.2 Side effects of sulfonylureas

The primary adverse effect associated with sulfonylureas is hypoglycemia. Due to variations in the pharmacotherapeutic properties of different sulfonylurea agents, the risk of hypoglycemic episodes can vary significantly among them (120, 134). The possibility of gaining weight is yet another drawback of sulfonylureas. Many people see an increase of at least 2 kg when taking these drugs (117, 135). It is also to be noted that certain people with sulfonamide allergies show cross reactivity with sulfonylureas; thus, these treatments shouldn’t be used in patients with sulfa allergies. Cross-reactivity with other medicines, such as carbonic anhydrase inhibitors, loop diuretics, and thiazide diuretics, is also possible (120, 136). Sulfonylureas are also linked to an increased risk of cardiovascular disease (68, 137). According to research, while promoting the closure of pancreatic-cell KATP channels to boost insulin secretion, this medicine may also lead to the closure of cardiac KATP channels, resulting in a higher cardiac risk in those people (138, 139).

### 3.4 Meglitinides

Nateglinide and repaglinide are the two most common meglitinides (glinides) (62, 140–142). The insulinotropic drugs are meglitinide analogues. They were introduced in 1995 and were licensed for clinical use in people with T2DM in 2000. They are secretory substances with a faster anti-hyperglycemic activity and a shorter duration of action than sulfonylurea. As a result, post-prandial hyperglycemia is better managed, and the risk of late hypoglycemia is also decreased (143–146).

#### 3.4.1 Mode of action

Meglitinides attach themselves to Sulfonylurea Receptor 1 (SUR1’s) benzamido site on β cells (123, 147). The first meglitinide analogue approved for clinical use in adults with T2DM was repaglinide. The insulinotropic effect of repaglinide, similar to that of sulfonylureas, operates through ATP-dependent potassium (KATP) channels. Repaglinide stimulates insulin secretion by inhibiting KATP channels in pancreatic β-cells, leading to membrane depolarization and the opening of voltage-gated calcium channels. The resulting influx of calcium increases intracellular calcium levels, triggering the exocytosis of insulin-containing granules (148, 149). Nateglinide, like repaglinide, binds to SURs, blocking KATP channels and promoting insulin secretion, but its pharmacodynamic effects are distinct in several important respects (150, 151).

#### 3.4.2 Adverse effects of meglitinides

Repaglinide and nateglinide studies have revealed different levels of hypoglycemia and, overall, lesser weight gain than sulfonylureas (149, 152–163). Although a topical test-positive delayed-type hypersensitivity reaction to repaglinide has been documented, cutaneous reactions to meglitinides seem to be uncommon. In this case, the fifth day after starting repaglinide, a maculopapular rash developed. Repaglinide was stopped, and systemic corticosteroids and antihistamines were administered instead (158, 164). Six people in a post-marketing monitoring study of nateglinide reported seven cutaneous side effects associated with treatment, including two occurrences of allergic dermatitis and one non-specific rash. There were 892 patients in the study overall (153, 165).

### 3.5 α-Glucosidase inhibitors

In the early 1990s, acarbose was the first alpha-glucosidase inhibitor to be introduced. Miglitol and voglibose also became available later on. It is common for Asian communities to utilize AGIs, especially those who consume diets that are high in complex carbohydrates (166, 167).

#### 3.5.1 Mechanism of action

In the brush border of enterocytes lining the intestinal villi, α-glucoside inhibitors (AGIs) competitively inhibit α-glucosidase enzymes, preventing the enzymes from cleaving disaccharides and oligosaccharides into monosaccharides (167–170). This process helps to reduce fluctuations in blood glucose levels and decrease the amount of insulin required during meals by slowing down the digestion and absorption of carbohydrates in the lower part of the digestive system (148, 167). Compared to controls, AGI therapy reduces Glucagon-like Peptide (GIP) secretion and increases postprandial Glucose-dependent Insulinotropic Polypeptide (GLP-1) secretion (171–173). Varying α-glucosidase enzymes have distinct affinities for AGIs, resulting in unique activity profiles. For example, acarbose shows a higher affinity for glucoamylase while miglitol is a more effective sucrase inhibitor (167, 174).

#### 3.5.2 Adverse effects of α-glucoside inhibitors

The common gastrointestinal side effects caused by AGIs, such as flatulence, stomach pain, and diarrhea, may lead patients to discontinue their medication (123, 175).

### 3.6 Dipeptidyl peptidase-4 inhibitors

The DPP-4 inhibitors currently on the market are sitagliptin, vildagliptin, saxagliptin, linagliptin, and alogliptin (176, 177). Japan has granted licenses for two DPP-4 inhibitors, omarigliptin and trelagliptin (178–181).

#### 3.6.1 Mechanism of action

DPP-4 inhibitors increase levels of incretin hormones in circulation, notably GLP-1 and GIP. The “incretin effect” refers to the ability of intestinal variables to increase insulin responses by 50-70% in healthy individuals following a diet (182–184). In T2DM, this effect is significantly reduced. When lipids and carbohydrates are consumed, K cells in the duodenum and jejunum release glucose-dependent insulinotropic polypeptide (GIP) (185–190). Apart from its incretin action, GIP also plays roles in adipogenesis and potentially β-cell proliferation, and reduces stomach acid output (188, 190–198).

#### 3.6.2 Adverse effects

There is an uncertain safety concern over the long run, which may raise the possibility of pancreatitis and an increased risk of liver dysfunction associated with Vildagliptin (123).

## 4 Plant-based metallic nanoparticles in diabetes management

As previously mentioned, the use of synthetic medicines for diabetes treatment is often hindered by their associated side effects (199, 200). Consequently, the current focus is on exploring the antihyperglycemic potential of medicinal plants in managing diabetes. According to worldwide ethnobotanical studies, approximately 800 plant species are employed for medicinal purposes in preventing diabetes (201, 202). Among these, scientific validation has confirmed that only 450 of these plants possess properties capable of lowering blood glucose levels, with 109 of them having well-documented mechanisms of action (58, 203).

Various treatments are also being employed in a holistic approach that takes into account physical, psychological, and spiritual aspects (204, 205). Notably, between 60% to 80% of the global population utilizes traditional medicines derived from medicinal plants to address various health conditions, including diabetes. There is a multitude of plants known for their anti-diabetic properties (206, 207).

### 4.1 Preparation and characterization of plant-based metallic nanoparticles

#### 4.1.1 Synthesis methods

Using green synthesis techniques, plant extracts serve as both capping and reducing agents in the synthesis of plant-based metallic nanoparticles (NPs). The extracts, which are high in alkaloids, terpenoids, phenolics, and flavonoids, are combined with metal precursors such as gold chloride (HAuCl_4_), zinc sulfate (ZnSO_4_), or silver nitrate (AgNO_3_) (208). The pH, temperature, and reaction time are all regulated during the biosynthesis, which is usually shown by a color shift brought on by surface plasmon resonance (for example, AgNPs turning from pale yellow to dark brown) For example, the aqueous leaf extract of *Musa paradisiaca* is frequently utilized as a stabilizing and reducing agent in the synthesis of zinc oxide nanoparticles (ZnONPs). The plant extract’s bioactive components, including flavonoids, polyphenols, and reducing sugars, convert Zn²^+^ ions to ZnO nuclei when the zinc nitrate hexahydrate [Zn(NO3)2·6H2O]^+^ precursor is introduced under carefully monitored conditions (usually 60–80°C, pH ~9). These phytochemicals also cap the developing nanoparticles as the reaction goes on, limiting aggregation and encouraging size control. A color shift (such as a yellowish-white precipitate) usually signals the creation of ZnONPs, and UV-Vis spectroscopy, which shows a distinctive absorption peak at about 360–380 nm, confirms this (209).

Similarly, *Argyria nervosa* root extract is used to synthesize silver nanoparticles (AgNPs) by reducing Ag^+^ ions from silver nitrate (AgNO_3_). The phytochemicals present, particularly phenolics and alkaloids, donate electrons to reduce Ag^+^ to metallic Ag⁰, initiating nanoparticle nucleation. Simultaneously, these compounds act as capping agents, forming a protective layer around each nanoparticle that improves colloidal stability and biocompatibility (210) (**Tables 2**, **3**). In these green synthesis systems, plant-derived compounds become physically or chemically associated with the nanoparticle surface. For crude extracts, the nanoparticles serve as nano-carriers, embedding or adsorbing various phytochemicals in their matrix or on their surface. This makes it possible for several bioactives, like enzyme inhibitors, anti-inflammatory drugs, and antioxidants, to be delivered simultaneously in a single nanoformulation (239).

**Table 2:** Antidiabetic action of plant-based AgNPs.

Plants sources	Mechanism action	Reference
*Argyeria nervosa* (leaves)	These secondary metabolites, carbohydrates, phenols, sterols, terpenoids, and flavonoids have applications in zerovalent NPs preparation. About 100 µg/ml caused the 70% inhibition of α-amylase and α-glucosidase enzymes.	(211–214)
*Callophylum tomentosum* (leaves)	About 500 µg/ml blocks the activities of α-amylase 18%, α-glucosidase 52%, and Dipeptidyl Peptidase-IV (DPPIV) 58%, by contribution of tannins, alkaloids, coumarins, glycosides, and flavonoids.	(215, 216)
*Cantella asiatica* (leaves)	These biochemicals, carbohydrates, glycosides, proteins, and alkaloids contributed to the NPs preparation. About 200µg/ml enhances the glucose uptake up to 63%, 53% in non-enzymatic glycosylation, while 44% decreases the α-amylase at 1000µg/ml.	(217, 218)
*Clausena anisate* *(roots)*	Alkaloids, terpenoids, and flavonoids are taking part. About 500µg/ml hang up 83.60% α-amylase and 10mM increase glucose uptake 69.51%.	(219, 220)
*Eysenhardtia polystachya* (barks)	Flavonoids. Alcohol and alcoholic derivatives compounds intermingled in the NPs synthesis. It decreases blood glucose level and enhances the insulin secretion by INS-1 at 10µg/ml.	(221, 222)
*Musa paradisiaca* (stem)	Alkaloids, saponins, glycosides, and steroids are involved in NPs preparation. It reduces the glucose level 281.08–208 mg/dl, and enhances the insulin level 16.12µU at 50µg/kg, and glycogen rises 38.51-29.42 mg/g, while the glycosylated hemoglobin is reduced to normal at 50µg/kg and elevates the hemoglobin.	(223, 224)
*Solanum nigrum* (leaves)	Alkaloids, saponins, tannins, flavonoids, and phytosteroids are contributed from a plant source. About 10mg/kg reduced the blood glucose level from 250mg/dl- 125 mg/dl and maintained the body weight.	(225, 226)
*Tephrosia tinctoria* (stem)	Flavonoids and phenols contribute to NPs preparation. 75µg/ml immobilized 83.52% α-amylase and 95% α-glucosidase activities, and enhanced the glucose level in hemoglobin by 1.19%.	(227)

**Table 3:** Antidiabetic application of ZnONPs.

Plants sources	Mechanism action	Reference
*Azadirachta indica* (leaves)	Flavonoids, phenolic compounds, saponins, and glycosides are involved. 100µg/ml concentration inactivates the α-amylase and α-glucosidase 85.7% and 87% respectively.	(228, 229)
*Sonneratia apetala* (leaves)	By the conjugation of carbohydrates, proteins, lipids, steroids, and cardiac glycosides, NPs are prepared. HF-ZnONPs and SA-ZnONPs, 334µg/ml and 394µg/ml, stop the activity of α-amylase.	(230, 231)
*Moringa oleifera* (leaves)	The mixture of proteins, flavonoids, glycosides, and tannins is involved. About 100µg/ml inactive the 90% α-amylase and 96% α-glucosidase.	(229, 232)
*Silybum marianum* (Seeds)	Flavonoids and silybin derivative compounds are conjugated with these NPs. 96-207mg/dl dose lowers the Fasting blood sugar while enhancing the insulin and High-Density Lipoprotein (HDL) level.	(233, 234)
*Urtica dioica* (leaves)	Palmitic acids, stearic acid, alkanes, and tetrachlorohydroquinone are contributed. HDLC and insulin level enhance 181% and 130% respectively, while they lower the level of TG, TC, and FBS 39%, 17.4%, and 51.7% respectively.	(235, 236)
*Vaccinium arctostaphylos* (flowers)	Flavonoids, polyphenols, and anthocyanins are involved. It reduced the fasting blood glucose from 175 to 50 mg/ml but had no impact on insulin.	(237, 238)

The production of gold nanoparticles (AuNPs) using extract from *Typha capensis*, which includes bioactive flavonoids and glycosides, is another well-researched example. The gold ion precursor in this method is chloroauric acid (HAuCl_4_). When the phytochemicals are combined with the plant extract and heated to about 70°C, they convert Au³^+^ to elemental Au⁰, which starts the nucleation of nanoparticles. The extract’s flavonoids stabilize the suspended nanoparticles by attaching to the gold surface and acting as capping ligands in addition to reducing agents (240). Additionally, certain bioactive substances, like naringenin, can be added to these AuNPs either post-synthesis or from the same extract. Naringenin finds adsorption sites on the surface of the nanoparticles through π-π stacking interactions or hydrogen bonding. Consequently, the plant-derived substance with enhanced cellular absorption and prolonged release is delivered by the nanocarrier system along with the metallic therapeutic activity (e.g., antioxidant, anti-inflammatory from AuNPs) (241).

#### 4.1.2 Characterization techniques

Analyzing the composition and functionality of synthesized NPs using key approaches includes:

##### 4.1.2.1 UV–Visible spectroscopy

Utilized to identify the surface plasmon resonance (SPR) peaks characteristic of metallic nanoparticles to verify the formation of nanoparticles. Peaks for silver nanoparticles typically show up between 400 and 450 nm (242).

##### 4.1.2.2 Fourier transform infrared spectroscopy

Determines the functional groups that are involved in NP stabilization and reduction. This validates how phytochemicals from plant extracts, such as -OH, -COOH, and -NH₂, play a part in NP capping and surface chemistry (243).

##### 4.1.2.3 Dynamic light scattering

Assesses the stability of colloidal particles in solution by measuring hydrodynamic size, zeta potential, and the polydispersity index (PDI) (244).

##### 4.1.2.4 X-ray diffraction

Determines the NPs’ crystalline structure. Specific crystalline planes are represented as peaks in XRD patterns, indicating that the particles are metallic (245).

##### 4.1.2.5 Scanning and transmission electron microscopy

Gives information on particle size distribution and morphology at the nanoscale. The particle shape (spherical, rod-like, etc.) that affects biological interactions is also revealed by TEM (246).

##### 4.1.2.6 Zeta potential analysis

Evaluates the surface charge to determine the stability of dispersion. Zeta potentials of ±30 mV or above indicate strong anti-aggregation stability (247).

#### 4.1.3 Drug loading strategies

From the reference to tables (2–6), plant-based metallic NPs in this review utilize:

##### 4.1.3.1 Crude extracts

The majority of formulations comprise entire plant extracts that contain many phytochemical components, such as alkaloids, flavonoids, and tannins (from, for example, *Solanum nigrum*, *Tephrosia tinctoria*, and *Eysenhardtia polystachya*) (248).

##### 4.1.3.2 Isolated compounds

Numerous studies used particular bioactive substances, including naringenin in AuNPs produced from *Typha capensis* or silybin from *Silybum marianum* (240).

#### 4.1.4 Administration routes and formulations

In the preclinical studies reviewed, the following administration routes were commonly reported:

##### 4.1.4.1 Oral administration

When it comes to administering plant-based metallic nanoparticles in antidiabetic models, this is the most popular method. As an example, ZnONPs produced from *Musa paradisiaca* were taken orally at a dose of 50 µg/kg, which markedly raised insulin levels and decreased blood glucose (**Table 2**). When taken orally at a dose of 10 mg/kg, NPs derived from *Solanum nigrum* extract showed a decrease in blood glucose and a maintenance of body weight in diabetic mice (**Table 2**). At 100 µg/ml oral dosages, *Moringa oleifera* ZnONPs demonstrated substantial inhibition of α-amylase and α-glucosidase (**Table 3**).

##### 4.1.4.2 Intraperitoneal injection

Used, especially in models of male infertility, to evaluate systemic effects on oxidative or hormonal markers. For example, when 200–1000 mg/kg of costus after AgNPs were injected intraperitoneally, the levels of serum testosterone, LH, and FSH rose (**Table 4**). To enhance sperm quality and antioxidant enzyme activity, diabetic mice were given injections of 2 mg/kg of *Withania somnifera*-derived SeNPs (**Table 4**).

**Table 4:** Plant-based metallic NPs for the treatment of male infertility.

Plant name	NPs	Concentration	Activity	Reference
*Costus afer*	Silver NPs from leaf extract	200mg/kg- 1000 mg/kg	Serum levels of testosterone, LH, and FSH significantly increased	(249)
*Withania somnifera*	Selenium NPs	2mg/kg	Increased antioxidant enzyme activities and improved sperm quality	(250)
*Moringa olifera*	Zinc Oxide NPs	10mg/kg	Improve testicular damage, Apoptosis, and Steroidogenesis-Related Gene Dysregulation.	(251)
*Panax ginseng*	Ginseng NPs emulsion	125 and 250 mg/kg	Improved level of testosterone	(252)
*Aloe barbadensis*	zinc oxide NPs	5mg/kg	Protective effects on testis histology, sperm parameters, oxidative stress markers Androgen production in rats treated with cisplatin	(253)
*Nigella sativa*	Silver NPs	1.5 mg/kg	Reduce hexavalent chromium toxicity, and toxic heavy metals cause harm to the reproductive system.	(254)
*Ocimum tenuiflorum*	silver NPs	1.5 mg/kg	fertility diagnosis	(255)
*Typha capensis*	GoldNPs AuNPs	1.5 mg/kg	Prostate cancer therapy	(240)

##### 4.1.4.3 Other routes

*Nigella sativa*-mediated AgNPs were orally administered in toxicity reversal studies involving hexavalent chromium exposure (**Table 4**).

##### 4.1.4.4 Therapeutic advantages of nanoparticle-based delivery

In a study employing ZnONPs made from *Moringa oleifera*, oral treatment at 100 µg/ml inhibited α-glucosidase by 96% and α-amylase by 90%, suggesting a potent postprandial glucose-lowering action with negligible liver damage (**Table 3**). Intraperitoneally administered costus after-derived AgNPs at 200 mg/kg significantly decreased testicular lipid peroxidation (MDA) levels and increased endocrine restoration with less oxidative stress, restoring testosterone levels from 2.1 ng/mL in diabetic controls to 4.5 ng/mL (**Table 4**). Because of their superior stability and targeted administration, quercetin-loaded AuNPs demonstrated a three-fold increase in cellular absorption in β-cells and decreased cytotoxicity to liver cells at equivalent therapeutic doses when compared to free quercetin.

#### 4.1.5 Synergistic mechanisms between plant phytochemicals and metal nanoparticles

Plant-mediated metal nanoparticles have synergistic therapeutic effects that result from the interaction of metal cores (such as Au, Ag, Zn, and Mn) with bioactive phytochemicals (like flavonoids, alkaloids, and phenolics) (208). In addition to improving the solubility and bioavailability of encapsulated plant components, metallic nanoparticles shield them against early degradation. To replicate natural pharmacokinetics, this permits sustained release. In diabetic and cancer animals, for example, AuNPs loaded with naringenin from *Typha capensis* demonstrated enhanced delivery. Metal nanoparticles (NPs) such as ZnO or AgNPs may reduce oxidative stress by upregulating endogenous antioxidant enzymes (e.g., SOD, CAT, GPx), while plant components frequently scavenge free radicals. By doing so, oxidative damage to cells decreases (240).

For example, in diabetic mice, SeNPs derived from *Withania somnifera* increased antioxidant defense and decreased ROS, improving sperm quality (250). The inhibition of important enzymes in glucose metabolism (α-amylase, α-glucosidase, and DPP-IV) by both phytochemicals and metal nanoparticles can result in additive or synergistic hypoglycemic effects. The metal ion activity and polyphenolic stabilization of *Moringa oleifera*-ZnONPs were responsible for the 90% and 96% inhibition of α-amylase and α-glucosidase, respectively. Furthermore, phytochemicals facilitate receptor-mediated endocytosis of NPs by acting as biocompatible capping agents. This improves insulin signaling and tissue regeneration by increasing NP uptake into target cells (such as pancreatic β-cells and Sertoli cells) (256). For instance, male rats’ levels of LH, FSH, and testosterone were raised by *Costus after-derived* AgNPs. This was probably because of increased bioavailability and hormonal signaling through NP absorption (257).

#### 4.1.6 Structural analogies and comparative efficacy of plant-based actives

The structure and functional characteristics of many active chemicals originating from plants are comparable to those of commercially marketed medications. For instance, thiazolidinediones and flavonoids like quercetin and kaempferol have structural similarities and can bind to PPAR-γ to increase insulin sensitivity (258). The same mechanism that metformin targets, AMPK, is also activated by berberine, and silybin from *Silybum marianum* has antioxidant and insulin-sensitizing properties similar to those of hepatoprotective drugs (259). In addition to sharing structural similarities with synthetic pharmaceuticals, these substances also modulate insulin signaling, glucose metabolism enzymes (including α-glucosidase and α-amylase), and reproductive hormone pathways.

Due to the synergistic effects of several phytochemicals acting on various targets at once, crude plant extracts frequently exhibit efficacy that is about equal to or even better than isolated substances. For example, formulations using extracts of *Azadirachta indica* and *Moringa oleifera* for ZnONPs showed over 85% inhibition of important enzymes involved in the digestion of glucose, which is comparable to that of common medications such as acarbose (260) (**Table 3**), whereas the extract from *Eysenhardtia polystachya* increased the release of insulin via INS-1 pancreatic cells (**Table 2**). Improved bioavailability, wider therapeutic coverage, and natural excipients that promote stability and solubility are all advantages of these multi-component systems (221). However, there are drawbacks to crude extracts as well, including unpredictability, ambiguity in composition, and standardization issues. Curcumin and naringenin, on the other hand, are single-component phytochemicals that provide superior control over nanoparticle formulations, promoting regulatory compliance and reproducibility (261). In general, plant-based bioactives serve as natural substitutes for synthetic medications, and both isolated molecules and crude extracts offer special benefits that can be used strategically depending on the intended therapeutic outcome (262).

Delivering therapeutic agents to their target sites presents several challenges, including poor bioavailability, *in vivo* instability, low solubility, limited absorption, target-specific delivery difficulties, and suboptimal therapeutic efficacy. To address these limitations, advanced targeted drug delivery systems have been developed. Among these, nanotechnology has emerged as a promising interdisciplinary approach, offering cost-effective and innovative solutions for precise drug delivery (263). In particular, metal nanoparticles (NPs) have gained significant attention due to their wide-ranging applications in medicine, biology, and physics, making them valuable tools for enhancing the efficiency and specificity of therapeutic interventions (263, 264). The use of plant-based metallic NPs in diabetes treatment offers a promising approach for the precise delivery of therapeutic compounds (**Table 2**). Derived from natural sources, these NPs are biocompatible and can be engineered to encapsulate insulin, antidiabetic medications, and plant-derived bioactive compounds. This allows for sustained and targeted delivery, mimicking the body’s insulin release patterns, regulating blood glucose levels, and potentially reducing the dosage needed. Additionally, these NPs can help mitigate diabetes-related inflammation and oxidative stress, protect against complications, and enhance the bioavailability of oral medications (265). These phytogenic nanoparticles offer various advantages compared to synthetic antidiabetic drug (**Table 5**).

**Table 5:** Comparative analysis of synthetic anti-diabetic drugs *vs*. plant-based metallic NPs.

Measures	Synthetic drugs	References	Plant-derived nanoparticles	References
Potency	1.8% HbA1c levels dropped (SGLT2 inhibitors)	(266)	89% α-amylase inhibition by Moringa oleifera-derived ZnONPs.	(267)
Onset	30–60 min effect of GLP-1 RAs.	(268)	12 min glucose reduction by Sargassum-derived AuNPs.	(269)
Adverse effects	4.2% genital infections with SGLT2i.	(270)	Renal clearance issues with AgNPs >20 nm	(271)
Expense	More expensive	(272)	Cost-effective for optimized green synthesis.	(273)
Process	SGLT2 activity inhibition	(273, 274)	Simultaneous activation of α-amylase and GLUT4	(43)
Fertility Outcome	Potential sperm DNA damage	(250)	40% motility increase with SeNPs.	(275)

Utilizing *Nigella sativa* plant extract, Alkhalaf et al. (276) Assessed the green prepared Ag NPs for diabetic neuropathy. A comparison between diabetic neuropathy-induced and stable control groups revealed a significant increase in blood glucose levels, advanced glycation end products (AGEs), and aldose reductase activity, accompanied by a marked reduction in insulin levels. Additionally, inflammatory markers were significantly elevated in the diabetic neuropathy group. Notably, nitrotyrosine levels were substantially lower, suggesting a notable alteration in the oxidative state. Gene expression analysis further demonstrated a significant upregulation of nerve growth factor (NGF) and a downregulation of brain Tyrosine Kinase Receptor A (TrkA) in individuals with diabetic neuropathy compared to healthy controls. Various therapeutic interventions targeting diabetic neuropathy have shown significant improvements across all evaluated biomarkers (276).

Cheng et al. (277) conducted a study involving the manufacturing of Ramulus Mori extract loaded on polyacrylic Gold nanoparticles (AuNPs) as an antidiabetic agent, specifically gestational diabetes mellitus nursing (GDM). Microscopic examination of the livers in diabetic mother rats revealed characteristic alterations in cell layers. However, administering Au NPs at the maternal stage significantly improved. Biochemical analysis indicated that Au–PAA–NPs contributed to the amelioration of alterations in maternal serum glucose concentration. Govindan et al. (278) created eco-friendly Zn-doped *Catharanthus Roseus* plants, aiming to enhance anti-diabetic properties through inhibiting alpha amylase activity.

A recent study revealed that *Allium cepa* extract-prepared NPs effectively suppressed the activity of α-amylase and α-glucosidase enzymes. Since α-amylase plays a crucial role in carbohydrate metabolism, inhibiting its activity is considered a promising strategy to lower blood sugar levels. Additionally, these amylase inhibitors act to reduce the absorption of dietary starch in the body. Likewise, α-glucosidase is a key enzyme in carbohydrate metabolism, facilitating the breakdown of disaccharides and oligosaccharides into monosaccharides (279, 280). Furthermore, NPs derived from plants and coated with phytochemicals, and other vital bioactive compounds sourced from plant secondary metabolites may find application in treating foot or limb infections in individuals with diabetes (281, 282).

Silver NPs derived from *Argyreia nervosa* and *Punica granatum* (211, 283), and marine algae *Colpomenia sinuosa* (284, 285) Have been documented for their antidiabetic properties. They exhibited a dose-dependent inhibition of α-amylase and α-glucosidase activities, as compared to the crude plant extracts. Moreover, silver nanoparticles (AgNPs) derived from the stem section of *Tephrosia tinctoria* were found to inhibit the activities of α-amylase and α-glucosidase, while also enhancing glucose uptake in human red blood cells (286, 287).

Shanker et al. (288) Reported that Zinc oxide nanoparticles (ZnONPs) derived from different plant sources such as *Momordica charantia*, *Azadirachta indica*, *Hibiscus rosa-sinensis*, *Murraya koenigii*, *Moringa oleifera*, and *Tamarindus indica* have antidiabetic potential by significantly inhibiting the activities of α-amylase and α-glucosidase(**Table 3**). The anti-diabetic properties of ZnONPs prepared from the leaves of the insulin plant (*Costus igneus*), by diminished the activities of α-amylase and α-glucosidase, were reported by (282). Gold NPs (AuNPs) obtained from *Cassia auriculata* (289) And *Sargassum swertzii* (Dhas et al., 2016) demonstrated antihyperglycemic action in rats after induced diabetes. Gold NPs have been found the application biocompatible, nontoxic, and easily interact with a variety of biomolecules such as amino acids, proteins, enzymes, and DNA, also playing a vital role in their immobilization (**Table 6**).

**Table 6:** Antidiabetic application of AuNPs.

Plants sources	Mechanism action	Reference
*Cassia auriculate* (Flowers)	Proponic acid conjugate with NPs, and 0.5µg/kg, balances the cholesterol, insulin, and body weight in diabetic induced rodents.	(289, 290)
*Cassia fistula* (stem bark)	Flavonoids, phenolics, and anthroquinones are involved in NPs preparation. It enhances the protein content, globulin, and serum albumin while reducing the alanine, aspartate transaminase, and alkaline phosphatase.	(291, 292)
*Gymnema sylvestre* (leaves)	Alkaloids, flavonoids, and terpenoids stabilized them and reduced the level of cholesterol, triglycerides, and LDL-C, and gave rise to HDL-C in rodents.	(293, 294)
*Saraca asoca*	Carbohydrates, proteins, saponins, and flavonoids make the zerovalent NPs. It immobilized the α-amylase enzyme.	(295, 296)
*Sargassum swartzii*	Alkaloids, flavonoids, steroids, and phenolic groups stabilized them. It enhances the insulin and HDL-C, and reduces FBS, TG, LDL-C, TC, and hemoglobin.	(268, 297)

Plant-based metallic NPs have been found to heighten antimicrobial resistance in diabetic patients even at lower concentrations, exhibiting lower toxicity to the human body (**Table 7**). These SNPs induce bacterial cell damage through two mechanisms. Firstly, they adhere to the bacterial cell wall, disrupting permeability and cellular respiration (298). Additionally, they cause damage by interacting with phosphorus- and sulfur-containing compounds like DNA and proteins (299). Based on the *Streptomyces achromogenes* bacterium, Streptozotocin (STZ) serves as a broad-spectrum antibiotic. Additionally, it functions as a pancreatic beta-cell–specific cytotoxin, making it a commonly used inducer of diabetes in rat models (280).

**Table 7:** Antidiabetic application of various phyto-NPs.

Plants source	Metal source	Mechanism action	Reference
*Gnidia glauca (*leaves)	Copper	Phenolics and flavonoids act as carriers. 100µg/ml concentration caused 88.6% immobilization of α-glucosidase.	(300)
*Ocimum basilicum* (flower)	Au-Ag composites	Flavonoids, phenolics, terpenes, and cyclohexane are involved in zerovalent NPs preparation. It reduced the activity of α-amylase by 70% and α-glucosidase by 78.62%, respectively.	(280, 301, 302)
*Stevia rebaudiana* (leaves)	Chitosan	Diterpenes glycosides are involved in NPs preparation. It normalized the biochemical assay and reduced the blood glucose level by around 167.2%.	(303)
*Zanthoxylum armatum* (Fruits)	Pd-rGO composite	Sterols, alkaloids, amino acids, glycosides, fatty acids, and benzenoids are involved in NPs synthesis. 0.0218µg/ml caused immobilization of α-glucosidase enzyme activity.	(304)

STZ elevates malondialdehyde (MDA) and nitric oxide (NO) levels while reducing the antioxidant capabilities of catalase (CAT), superoxide dismutase (SOD), GR, and glutathione peroxidase (GPx) in rodents. It utilizes specific receptors such as glucose transporter-2 (GLUT 2) receptors, competing with glucose molecules and leading to AKt phosphorylation, also referred to as protein kinase B. Moreover, STZ triggers apoptosis and cytotoxicity by elevating reactive oxygen species (ROS) and nitric oxide synthase production. It instigates oxidative stress, resulting in lowers testosterone levels, mitochondrial breakage, and DNA destruction through diminishing the antioxidant capacity of CAT, SOD, and other factors. Eventually resulting in the demise of cells. Through the utilization of receptor-mediated endocytosis for internalization, phyto-fabricated metallic NPs reduce the generation of ROS and nitric oxide synthase by boosting the antioxidant capacity of CAT, POD, serum testosterone, and lipid levels in STZ-treated rodents (305).

Furthermore, the synthesis of metallic NPs from plant extracts adds another dimension to the therapeutic potential of these natural remedies. NPs exhibit diverse shapes and sizes, which can influence their pharmacokinetics, biodistribution, and interactions with biological systems. For instance, NPs with smaller sizes may have enhanced cellular uptake and bioavailability compared to larger particles. Additionally, their unique shapes can affect their stability, surface area, and binding affinity to target molecules.

**Figure 3:**
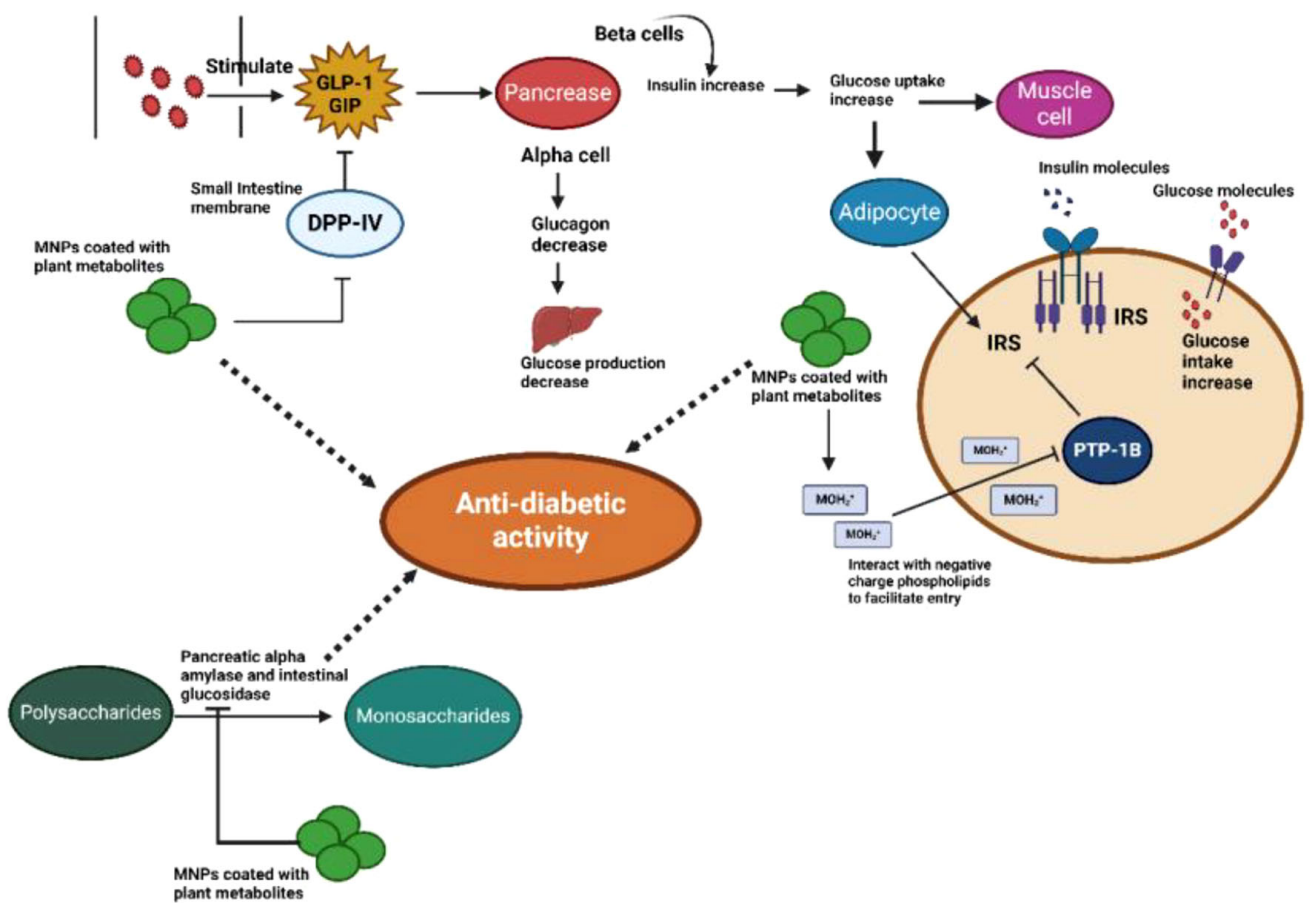
Anti-diabetic activity of plant-based metal NPs: The therapeutic target of monomeric plant peptidase IV (DPPIV), which is implicated in glucose regulation, is MNPs coated with plant metabolites in type 2 diabetes. When glucose or other nutrients are consumed, the intestine secretes two main incretin hormones that trigger the pancreatic β cells to secrete insulin: gastric inhibitory polypeptide (GIP) and glucagon-like peptide-1 (GLP-1). MNPs cause the serine protease enzyme DPPIV to be inhibited, as well as GLP-1 and GIP, which in turn causes an increase in the amount of insulin secreted by pancreatic β-cells. By inhibiting the PTP-1B enzyme on the ER membrane, MNPs stimulate insulin signaling pathways, which in turn cause adipocytes and muscle cells to absorb glucose. MNPs decrease the release of glucose by inhibiting the α-amylase and α-glucosidase enzymes.

When metallic NPs derived from plant extracts are used as antidiabetic agents, their distinct properties contribute to their efficacy through multiple mechanisms. These mechanisms may include modulating insulin sensitivity, enhancing glucose uptake by cells, inhibiting carbohydrate-digesting enzymes, and protecting pancreatic beta cells from oxidative stress. The specific combination of phytochemicals encapsulated within NPs, along with their size and shape characteristics, determines their overall impact on different regulatory pathways involved in diabetes management (**Figure 3**).

## 5 Plant-based metallic NPs for the treatment of male infertility

The use of plant-based metallic NPs for the treatment of diabetes-induced male infertility offers numerous potential advantages. They can be customized to deliver bioactive compounds to the male reproductive system, potentially enhancing sperm quality and motility. These NPs may also address inflammation associated with diabetes-induced male infertility. Furthermore, the encapsulation of plant-derived compounds can help regulate blood sugar levels, indirectly improving fertility and reducing side effects (306).

With a prevalence ranging from 2.5% to 12%, male infertility impacts more than 30 million men globally. It stands as the primary cause in 20%-30% of cases and contributes significantly to half of all instances of infertility (307–309). The key determinants of male fertility, including sperm count, quality, motility, and morphology, play crucial roles, and deviations in any of these factors can result in infertility (310). A substantial proportion, up to 90%, of infertile couples contend with issues related to low sperm count and/or poor sperm quality (257). Several factors, such as testicular development, reproductive system diseases, elevated scrotal temperature, immune system and endocrine disorders, as well as lifestyle choices, environmental conditions, and nutritional factors, have been identified as detrimental to sperm parameters, contributing to male infertility (251).

A significant proportion of male infertility cases remain idiopathic, highlighting the ongoing difficulty in determining definitive underlying causes. In such cases, the absence of a clearly defined etiology limits the availability of targeted pharmacological treatments. As a result, clinicians often resort to various empirical strategies aimed at stimulating spermatogenesis, which frequently produce inconsistent and non-standardized outcomes. In some countries, the off-label use of selective estrogen receptor modulators (SERMs), such as tamoxifen and clomiphene citrate, has been explored as a potential therapeutic option for managing male infertility (311). However, it is noteworthy that some drugs commonly used in this context have been linked to side effects that may impact male fertility. Within the realm of traditional medicine, plant-based preparations, including decoctions, concoctions, macerations, or infusions, are frequently employed to address a broad spectrum of ailments. Some of these botanical remedies are specifically utilized in connection with male reproductive health issues, acknowledging the global significance of these concerns as a public health and social challenge (312).

In recent decades, nanotechnology has emerged as a promising avenue for addressing infertility. NPs, characterized by their extremely small size (one billionth part of a meter - 10^-9), offer innovative solutions. While the chemical method of nanoparticle synthesis poses potential harm to human health and the environment, the biological method stands out as an eco-friendly, cost-effective, and reliable alternative. Nanostructures find diverse applications in gene delivery, tissue engineering, drug delivery, biological labeling, protein tracing, pathogen detection, cancer therapy, DNA structure analysis, and serve as contrast agents in magnetic resonance imaging (MRI) and molecular sensing. The green synthesis approach, employing plant extracts, presents notable advantages, including simplicity, cost-effectiveness, environmental friendliness, and reliability (249).

In a recent study by Egbiremhon et al. (313), the focus was on evaluating the impact of Costus afer-AgNPs extracts on male reproductive hormones in rats. The primary aim was to investigate the effects on testosterone, luteinizing hormone (LH), and follicle-stimulating hormone (FSH). The experimental groups, administered varying doses of 200mg, 400mg, 600mg, 800mg, and 1000mg of Costus afer-AgNPs extract per kilogram of body weight, demonstrated a significant elevation in serum levels of testosterone, LH, and FSH compared to the control group (P < 0.05). Remarkably, Costus after-AgNPs not only sustained but also exhibited the potential to augment the concentrations of these crucial reproductive hormones.

In their study, Ali et al. (250) Utilized a secure and non-hazardous approach to synthesize selenium nanoparticles (Se NPs) with the aqueous extract of *Withania somnifera* roots. The investigation focused on evaluating Se NPs’ potential to enhance antioxidant enzyme function and mitigate DNA damage in sperm, specifically in STZ-induced diabetic mice. The results demonstrated that Se NPs treatment increased antioxidant enzyme activities, improved sperm quality, and stabilized ROS levels in diabetic mice. The green synthesis method using plant extracts emerged as a secure means of producing Se NPs, with Se NPs exhibiting greater benefits compared to both inorganic and organic selenium counterparts.

Mostafa-Hedeab et al. (251) Conducted an assessment of the potential protective role of zinc oxide NPs synthesized via a green method using *Moringa oleifera* leaf extract (MO-ZNPs) against acrylamide (ACR)-induced reproductive dysfunctions in male rats. The results conclusively demonstrated the protective impact of MO-ZNPs, shielding male rats from ACR-induced reproductive toxicity. This effect was attributed to the suppression of oxidative injury and apoptosis, along with an augmentation in steroidogenesis and sex hormones. In summary, MO-ZNPs emerged as a valuable intervention to mitigate the adverse reproductive effects induced by ACR in male rats.

In their 2019 study, Kamel et al. (252) Addressed the clinically significant testicular toxicity associated with methotrexate (MTX). While previous research indicated ginseng’s potential to stimulate spermatogenesis and prevent chemotherapy-induced testicular injury, the study focused on formulating ginseng into NPs due to its low bioavailability. With limited available data on the protective effects of ginseng or its NPs against MTX-induced testicular toxicity, the findings of this study suggest that both ginseng and ginseng NPs protect against MTX-induced testicular toxicity in rats. This protective effect is attributed to the inhibition of MTX-induced testicular apoptosis, with ginseng NPs exhibiting a superior protective effect compared to ginseng at the given doses.

In their 2023 study, Nauroze et al. (254) aimed to investigate the adverse effects on the reproductive system induced by hexavalent chromium (Cr (VI)) and explore potential ameliorative effects using Nigella sativa and Nigella sativa-mediated silver NPs (AgNP) in male mice (Mus musculus). Clomiphene citrate, a known infertility medication, served as a positive control. The primary objective was to assess the ameliorative potential of orally administered substances, including 50 mg/kg body weight clomiphene citrate (control), chemically synthesized AgNP, *Nigella sativa* seed extract, and *Nigella sativa*-mediated AgNP, against the toxic effects of Cr (VI) induced by oral administration of K2Cr2O7 at a dose of 1.5 mg/kg body weight over eight weeks. The administration of Nigella sativa and Nigella sativa-mediated AgNPs demonstrated a reduction in toxicity.

The study by Jha et al. (255) Delves into nanocarrier-mediated targeted delivery and biosensing in reproductive health care. It specifically highlights the encapsulation of silver NPs (AgNPs) from *Ocimum tenuiflorum* within multiwalled carbon nanotubes (MWCNTs). This approach demonstrates the composite’s efficacy in targeting the intracellular region of sperm cells, suggesting its potential applications in biosensing-based infertility diagnosis. The investigation also confirms the binding and targetability of AgNP to the sperm nucleus, supported by assessments of DNA fragmentation and morphological examinations. The enhanced targeting efficiency and biosensing capabilities position the AgNP-MWCNT composite as a promising candidate for fertility diagnosis applications.

In their 2023 study, Pearce et al. (240) Explore the biomedical applications of green nanotechnology in addressing the challenges associated with the clinical use of naringenin, a flavone recognized for its emerging anti-cancer properties. Naringenin is naturally found in Typha capensis, a South African plant used in traditional medicine. Despite promising *in vitro* results, the study addresses limitations such as poor oral bioavailability and rapid metabolism. The research focuses on a novel drug delivery approach, reporting the successful synthesis of self-stabilized gold NPs (AuNPs) derived from naringenin. This innovation introduces an effective drug delivery tool with anticipated applications in prostate cancer treatment, aiming to enhance the delivery of anticancer therapeutics, particularly naringenin.

In a study, Majd et al. (253) Address the adverse effects of cisplatin (CP) on male reproductive tissues during cancer treatment. While the potential benefits of zinc oxide NPs (ZnO NPs) in cancer therapy have been extensively explored, limited data exists on the protective effects of green ZnO NPs against CP-induced male reproductive dysfunctions. The research involved comprehensive analyses, including testis histology, sperm parameters, oxidative stress markers, testosterone concentration, and the expression of genes related to steroidogenesis in different experimental groups. The findings indicate that green ZnO NPs exhibit notable protective effects, mitigating testis tissue damage and epididymal sperm disorders induced by CP. Across various factors, green ZnO NPs demonstrated a more potent protective effect compared to other forms of ZnO, suggesting their potential in attenuating CP-induced male reproductive dysfunctions (**Table 4**).

## 6 Challenges and considerations

Use of phytogenic metal NPs for treating diabetes and male infertility presents several challenges and considerations, particularly regarding safety and toxicity. Plant-based metal NPs must demonstrate biocompatibility to prevent eliciting immune responses or adverse reactions within the body, which could potentially lead to inflammation or tissue damage.

### 6.1 Unresolved toxicity profiles

While green-synthesized nanoparticles have demonstrated significant therapeutic potential in laboratory-based research, their unverified toxicological patterns remain a significant risk. Previous studies suggested that such nanoparticles can be involved in cytotoxicity, oxidative stress, and genotoxic alterations within biological systems (314). These nano-formulations have been associated with disruption of cellular architecture, metabolic imbalances, and induction of necrosis (315). Prolonged exposure and systemic accumulation can lead to chronic toxicity, necessitating comprehensive evaluations through longitudinal studies (314). As indicated by research findings that approximately 60% of the administered (AgNPs) accumulated in the liver and spleen of rodents within 28 days (316). Likewise, zinc oxide nanoparticles (ZnONPs) at doses higher than 50 mg/kg were found to induce oxidative stress in the testes of diabetic rats (251).

Assessing the long-term safety of phytogenic NPs is essential, particularly in chronic conditions such as diabetes and male infertility, where prolonged treatment may be necessary. Conducting longitudinal studies becomes essential to thoroughly evaluate any potential cumulative effects and chronic toxicity that may arise from prolonged exposure to these NPs. Understanding the interaction of phytogenic NPs with biological systems is essential for predicting their effects and potential toxicity. This includes studying their pharmacokinetics, biodistribution, metabolism, and excretion pathways. Phytogenic NPs are typically derived from plant-based compounds, which may have their safety profiles and toxicity concerns. Some phytochemicals may exhibit dose-dependent toxicity or interactions with medications. Assessing their potential to induce cytotoxicity, genotoxicity, or immunotoxicity is crucial. While phytogenic NPs hold promises for treating diabetes and male infertility, addressing safety and toxicity considerations is paramount for their successful clinical translation.

### 6.2 Lack of standardized synthesis protocols

Green synthesized formulation of nanoparticles utilizes diverse techniques notably eco-friendly synthesis routes involving botanical extracts. Developing standardized, reproducible, and scalable synthesis protocols is essential to achieve uniformity and reliability in therapeutic applications (317).

However, the field at the current stage lacks standardized production (317, 318). Factors such as plant species, extraction methods, climate, and reaction conditions can significantly affect the properties and efficacy of the nanoparticles. Aspects like plant taxonomy, extraction techniques, climate, and reaction parameters highly influence the properties and effectiveness of the resulting nanoparticles (318). Natural inconsistencies in raw materials further obstructed the standardization of plant-derived nanoparticles. For instance, shifts in climatic patterns alter the phenolic concentrations in *Argyreia nervosa* leaf extracts, thereby affecting the efficiency of AgNP biosynthesis (212). To validate reproducibility, protocols must include characterization via dynamic light scattering (DLS) and Fourier-transform infrared spectroscopy (FTIR) to confirm stability and surface functionalization of nanoparticles.

### 6.3 Insufficient human trial data

Although experimental research shows the potential of plant-derived nanoparticles in addressing diabetes and male infertility, verification from human trials remains limited (319). Most experimental work has relied on *in vitro* or animal models, restricting it to directly applying these results to human treatment without strong clinical validation (19). Comprehensive human studies are important for exploring how these nanoparticles behave in the body, including their distribution, absorption, metabolism, and possible long-term effects (319).

Robust preclinical and clinical studies are needed to evaluate their efficacy, safety, and long-term effects in patients. Furthermore, the personalized design of plant-based metallic NPs has the potential to significantly advance precision medicine techniques for the treatment of male infertility and diabetes. Utilizing the special advantages of plant-based substances and customizing nanoparticle compositions to match the specific needs of each patient, personalized NPs present a viable way to enhance therapeutic results while lowering risks and enhancing patient safety and satisfaction.

## 7 Conclusion

In conclusion, diabetes mellitus poses a substantial threat to male reproductive health, affecting critical functions such as ejaculation, erectile performance, reproductive organ integrity, and overall semen quality. While conventional synthetic anti-diabetic medications are widely used, their associated side effects and limitations underscore the urgent need for safer and more effective alternative therapies. Among emerging solutions, plant-derived metallic nanoparticles (NPs) have shown promising antidiabetic potential. These NPs have demonstrated the ability to modulate key regulatory pathways, including the inhibition of pancreatic α-amylase and intestinal α-glucosidase, enhancement of insulin activity, and improved glucose uptake. Such advancements offer considerable promise in addressing the complex interplay between diabetes and male infertility. Although preclinical studies have yielded encouraging results, clinical validation through Phase I/II trials is essential to confirm their safety and efficacy in humans. One promising strategy involves the repurposing of FDA-approved phytochemicals such as curcumin formulated into gold nanoparticles (AuNPs), which may streamline the regulatory approval process. To successfully transition these therapies from the laboratory to widespread clinical use, strong collaboration between nanotechnologists and healthcare professionals is crucial. Furthermore, the development of personalized, plant-based nanoparticle formulations represents a forward-thinking approach in precision medicine, offering tailored treatments for individuals affected by diabetes and infertility. Ultimately, these innovations hold the potential to significantly enhance human health and quality of life. While this review focuses on male infertility associated with diabetes mellitus due to its high prevalence and the abundance of relevant studies, it is important to note that diabetes also significantly affects female reproductive health, including menstrual irregularities, polycystic ovary syndrome (PCOS), and reduced fertility. Future research should explore the potential of plant-based metallic nanoparticles in addressing diabetes-related female reproductive disorders.
